# Sensemaking at work: How rationalized knowledge hiding and communication patterns affect decision quality

**DOI:** 10.1371/journal.pone.0353567

**Published:** 2026-07-17

**Authors:** Zhihao Gao, Muhammad Waseem Bari, Sadia Shaheen

**Affiliations:** 1 School of Economics and Management, Quanzhou University of Information Engineering, Quanzhou, China; 2 Lyallpur Business School, Government College University, Faisalabad, Pakistan; Westminster International University in Tashkent, UZBEKISTAN

## Abstract

This paper employs sensemaking theory to examine the relationships between rationalized knowledge hiding, communication patterns, and the quality of decision-making in information technology firms. Additionally, this study operationalized sensemaking processes as a mediator in the above mentioned relationships. The study employed a three-time lag approach with a 45-day gap, utilizing data from 348 managers, supervisors, and leaders. Partial least squares structural equation modeling and SmartPLS 4 are employed for the analyses. The results indicate that rationalized knowledge-hiding and communication patterns have a significant impact on decision-making quality. Moreover, sensemaking processes partially/complementary mediate the relationship between rationalized knowledge hiding, communication patterns, and decision-making quality. Incorporating sensemaking processes into the study model provides deeper insight into managers’ decisions and maps the temporal nature of these relationships. These valuable findings shed light on new meanings and directions for theoretical perspectives and their relevance in management and organizational behavior.

## Introduction

Decision-making quality (DMQ) remains one of the foundational pillars of success in every aspect of a person’s life, ranging from business ventures to simple daily decisions. Efficient and effective decision-making plays a crucial role in determining the success or failure of a firm. DMQ refers to the efficiency and effectiveness through which decisions are made, ensuring that such matters yield the most favorable results [1]. The key elements of DMQ are decision context, creative alternatives, helpful information, clear values, and logical and systematic thinking [1,2]. Despite the significance of decision-making, the mechanisms underlying DMQ are still complex and poorly understood. In decision-making, a set order is always followed, with logical reasoning and problem-solving through the evaluation and selection of the best alternatives. The DMQ is influenced by such factors as data utilization, heterogeneity of participants, and decision-making roles and responsibilities [3].

Rationalized knowledge hiding (RKH) is defined as the strategic and intentional concealment of knowledge requested by another person, and presenting the reasons behind the knowledge concealment [4]. RKH involves intentionally withholding information while providing a plausible justification. It is selective, strategic, and framed as legitimate (e.g., confidentiality, relevance, or timing). By contrast, evasive hiding or “playing dumb” relies on deception or feigned ignorance without justification and tends to erode trust and collaborative decision-making. RKH is an active approach where people purposefully retain knowledge for strategic motives [5]. The outcomes of RKH are multidimensional. RKH affects the immediate task performance and the long-term relational dynamics within groups [6]. Workers involved in RKH can strategically manage information flow, leading to greater effectiveness and a higher DMQ. The communication patterns (CPs) also help improve the organization’s DMQ, as all workers are informed and can contribute. Minimizing confusion and conflict leads to more united and informed decisions. Developing communication channels that welcome different opinions enhances the DMQ, as it incorporates a variety of solutions. The communication strategies, *i.e.,* regular team meetings, open communication channels, feedback mechanisms, and group decision support systems, can improve DMQ.

On the other hand, effective RKH management can lead to the selective revelation of information and the rationalization of sense-making processes (SMPs) by focusing on relevant information. The twofold nature of RKH underscores the importance of understanding its role in firm performance [7]. Reliable CPs are crucial for supporting SMPs within organizations. Open communication in the workplace is a process that enables workers to express their ideas and ask questions, establishing a shared reference point for approaching and understanding specific organizational issues. Cultivating positive communication processes is proposed to support sensemaking, as the richness of context-aided communication facilitates SMP. The overall quality of communication*, i.e., clarity, frequency, and openness, is expected to be* positively related to the SMPs. The SMPs can play a crucial role in enhancing DMQ. Employees with a practical sensemaking ability can better understand in-hand information and strengthen their DMQ. In other words, SMPs can positively impact DMQ by supporting individuals in constructing logical and actionable explanations of complex and uncertain situations.

[8] presented sensemaking theory as a logical approach to understanding how people understand and respond to complex and uncertain conditions. Sensemaking is an active process that enables individuals to derive meaning from their experiences and navigate unpredictable situations. Concerning RKH, sensemaking theory provides a framework for understanding how employees make sense of the information they receive and the gaps created by hidden knowledge. When knowledge is not shared strategically, employees will rely on the existing SMP available to them to develop a reconstructive understanding of their operating environment. Effective SMP can moderate the negative angle of RKH by enabling individuals to understand information and make informed decisions [9]. RKH shapes sensemaking by limiting which cues teams receive and framing omissions as justified. This selective information shifts how members interpret situations, often narrowing shared understanding. As a result, decisions may become faster but less accurate, since teams rely on incomplete cues and build explanations that overlook critical alternative interpretations.

Despite previous research considering knowledge hiding as a homogeneous, harmful process, few studies have investigated the concept of RKH and its dynamics in relation to communication patterns that influence interpretation and decision-making. This paper discusses this gap, reframing RKH as a potentially strategic driver of sensemaking, incorporating communication patterns as a contextual driver that alters information flows, and testing sensemaking as a mediator through a three-wave, time-lagged survey of IT managers and PLS-SEM analysis. The study narrows the scope of sensemaking theory by unraveling both direct and indirect impacts, offering a finer understanding of knowledge hiding, and providing managers with practical suggestions for the real world. This paper proposes that strategically managed RKH and CPs can positively affect DMQ and SMPs. This study has three research objectives. First, by examining the positive sides of RKH, this study aims to highlight RKH’s potential to streamline and enhance DMQ. Second, to underscore the importance of CPs in enhancing the DMQ. Third, to highlight the role of SMP as a mediator between RKH, CPs, and DMQ. This study has two research questions to achieve its objectives. First, what is the impact of RKH and CPs on DMQ? Second, how does SMP mediate the relationship between RKH, CPs, and DMQ? To provide empirical evidence, this study sourced data from IT firm managers and supervisors.

## Literature review

### Rationalized knowledge hiding and decision-making quality

The phenomenon of knowledge hiding, particularly RKH, has garnered the interest of scholars in organizational behavior and knowledge management [10,11]. RKH entails withholding information to protect confidentiality or other interests [12]. RKH, compared to other forms of knowledge hiding, such as evasive hiding and playing dumb, is strategic, knowledgeable, and purposeful, rather than detrimental to the organization’s performance [13]. Connelly et al. (2012) reveal that when employees conceal information based on rationality, decision-making becomes more deliberate as the scope of the decision is limited and possible bias is eliminated [14].

The RKH has a positive impact on DMQ in various ways. RKH can help improve DMQ by forcing users to focus on only the most relevant information. RKH can enhance DMQ by focusing attention on the most pertinent data. Drawing on sensemaking theory [8], individuals can alleviate cognitive burden by intentionally hiding peripheral data, allowing decision-makers to focus on central details [15]. Similarly, RKH helps protect sensitive information. In organizations where confidentiality is essential, RKH protects information. Such protection ensures that the firm does not experience data loss and maintains the integrity and quality of the decision-making process. [4,16]. Scholars also observed that when an organization or a peer keeps information out of employees’ reach, employees are likely to think for themselves. This can lead to creative ideas and an enhanced DMQ, as existing information cannot restrict people [17].

[18] explored the consequences of knowledge hiding. They found that RKH did not evoke negative emotions like guilt or shame, which are often associated with other forms of knowledge hiding (evasive or playing dumb). RKH can be a strategic tool for enhancing DMQ without the adverse effects typically associated with knowledge hiding. Similarly, [19] concluded that RKH fosters a creative culture that positively impacts DMQ. Employees’ independent thinking brings innovative solutions. [20] confirmed that RKH mitigates counterproductive behaviors, such as workplace incivility, thereby creating a more favorable environment for high DMQ. Considering these theoretical underpinnings and empirical investigations, this study proposes that.

**H1:** RKH has a positive impact on DMQ

### Communication patterns and decision-making quality

Communication processes concern the orderly manner in which information flows within an organization [21]. The CPs can be structural and non-structural, vertical or horizontal, and may be synchronous or asynchronous [22]. Effective communication procedures facilitate the transmission of information, prevent conflict, and promote cooperation. All of these are important for better DMQ [23,24]. Effective business communication is a crucial factor in organizational decision-making processes. Communication refers to the exchange of ideas between two or more people; it is an essential factor that outlines the methods and ways people interact concerning decisions [25]. Drawing on sensemaking theory [8] CPs are part and parcel of the sensemaking process. CPs are the media that transmit the information, the medium in which the data is analyzed, evaluated, and responded to. CPs enable the meaning to be jointly negotiated and the interpretations of a situation to be aligned.

Different CPs contribute to the construction of organizational identities, which in turn influence how individuals process information and make decisions. For instance, transparency in communications promotes a kind of social identity based on mutual trust, which in turn fosters greater cohesiveness within DMQ [26]. Organizational sensemaking is a process that entails how an organization understands prior events and makes contemporary decisions [9]. CPs include daily debriefs and feedback sessions because they allow for reflection, where others can discuss and gain knowledge from previous behaviors or practices [27]. Several empirical investigations have demonstrated that the right CPs have a positive impact on DMQ. For instance, a survey found that organizations with open communication channels and participatory decision-making mechanisms experienced higher DMQ and employee satisfaction [23]. Similarly, [28] explained that team communication should be as consistent as possible, allowing decisions to be made on time and increasing the likelihood of accuracy.

A clear, coherent communication structure enhances the organization’s ability to make sense of a crisis and to make the right decisions [9]. [29] indicated that more communicative middle managers are more effective in implementing strategic change. Effective vertical CPs enhance the extent of strategic fit and operational DMQ [30,31]. On the other hand, better horizontal CPs indicate higher innovation and better DMQ [32,33]. The mode of communication also impacts DMQ. Synchronous communication, such as real-time communication and the use of words to explain an issue, increases accuracy and DMQ. On the other hand, asynchronous communication, such as emails and memos, is more personal and detailed, allowing sufficient time to craft an appropriate response [34]. Effective CPs are integral to enhancing the DMQ within organizations. Organizations can improve their decision-making process, DMQ, and performance through effective, reciprocal, and open communication. Hence, this paper proposes that

**H2:** CPs have a positive impact on DMQ

### Sensemaking processes as underlying mechanism

The processes by which individuals come to understand information that is either unclear or difficult to comprehend, and use this understanding to direct their behaviors, are known as SMPs [8,9]. In organizational contexts, SMPs enable individuals or groups to anticipate the consequences of their actions within an organization, even when sufficient data is not available [9,35]. SMPs play a crucial role in bridging the gap between knowledge hiding and DMQ. RKH challenges employees to make high-quality decisions despite incomplete information or selective knowledge revelation [11,36]. In this scenario, SMP helps to moderate these challenges by enabling employees to reconstruct and interpret the existing information more effectively [8]. The DMQ is influenced by the availability and integrity of knowledge, as well as the capability to understand and apply it correctly [37].

SMP enables employees to understand fragmented or hidden information more successfully [9,38]. In the case of RKH, SMP helps persons restructure the missing pieces and develop logical insights from inadequate data [9,38]. This improved interpretation can lead to better DMQ, as employees can use existing information in their decision-making. [39] explained that the employees engaged in SMPs can better navigate the uncertainties caused by knowledge gaps and make better quality decisions [37,40]. This evidence suggests that SMPs can positively mediate the relationship between RKH and DMQ by providing a framework for interpreting and integrating existing information.

SMPs help individuals and teams decode communicated information, enabling them to make better-informed decisions. Scholars examined the role of CPs in DMQ and established that effective CPs, characterized by clear, frequent, and open exchanges, increase SMPs. SMPs, in turn, positively improve DMQ. Scholars claim that better communication enables better SMPs, leading to higher DMQ [27,41]. Similarly, authors found that CPs impact SMPs, which in turn mediate their effect on DMQ, highlighting the importance of designing communication approaches that support SMP to improve DMQ [42,43]. Based on several case studies and interviews, the authors confirmed that firms that support open communication and dialogue facilitate better sensemaking, which in turn helps improve DMQ [44,45]. In virtual teams, communication is usually facilitated by technology. Effective CPs in a virtual workplace help SMP, improving DMQ [46,47]. [48] examined the role of SMP in team-level decision-making. The findings confirmed that SMPs are essential for translating CPs into effective team decisions [49]. Grounded in the sensemaking theory and literature discussed above. The proposals are as follows,

**H3:** SMPs mediate the relationship between RKH and DMQ

**H4:** CPs mediate the relationship between RKH and DMQ

**Fig 1:**
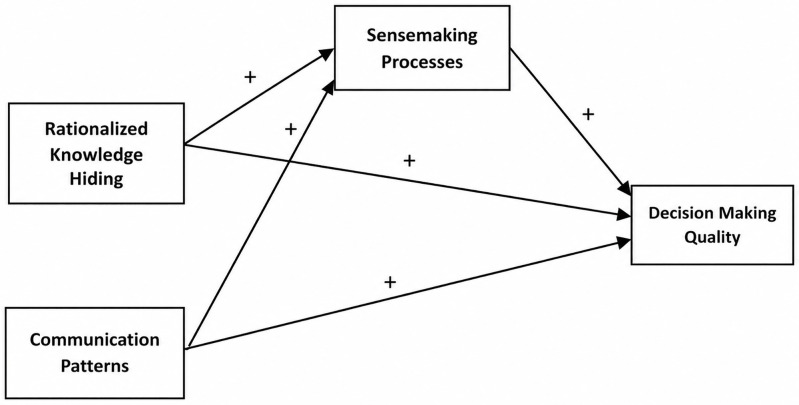
Study model.

Fig 1 presents the study structure and the relationships between variables.

## Research methods

### Data collection and its sampling

This study employs a primary data collection approach using a survey-based method. This study focuses on DMQ. Therefore, the authors collected the data from managers/supervisors working in information technology (IT) firms. The data were collected online using Google Forms. The questionnaire was designed in English because it is widely used. The RKH, CPs, SMP, and DMQ phenomena do not belong to specific contexts such as state, culture, or industry [50,51]. Such problems are common in organizations. Hence, the study targeted the official pages of IT firms on Facebook, WeChat, and LinkedIn to obtain multicultural and multi-organizational responses, thereby enhancing the generalizability of the results.

After consulting the administrators of social media groups and pages of IT firms, the authors selected 19 official pages based on two specific conditions. First, every official page must have at least 500 members. Second, the page administrator confirmed that many members are posted as supervisors or managers in the firm. It was an option for all employers, supervisors, and managers on the 19 pages (who are proficient in English) to participate in this study survey. Since most of these pages are set to private, the web link of the questionnaire was only posted on the 19 pages after the respective admins approved it. Short descriptions/guidelines were also added at the instrument’s start. In this description, the authors enumerated the research’s primary goals, the anonymity of participants, and the guarantee of participants’ information security. The participants were informed in this letter that the collected data would be used solely for the analysis of this particular study. Therefore, all respondents should feel relaxed and at ease when responding to surveys used in the study. To the study participants, it was explained that there was no pass or fail; instead, their perception and experience of the phenomenon were of interest. These descriptions help in minimizing what is referred to as social desirability bias [52].

Ethical approval was not required for this study. The research involved anonymous questionnaire responses from competent adults about perceptions of business practices and did not include physical interventions, biological samples, or sensitive personal data. In accordance with the Declaration of Helsinki and applicable institutional guidelines, anonymized, low-risk perception surveys are exempt from formal ethics committee review. Participation was voluntary, and informed written consent was obtained from all participants. A written consent statement was provided on the cover page of the survey instrument and shared through the online survey weblink; participants indicated consent before proceeding to complete the questionnaire.

Data were anonymized at the point of collection and stored securely. The study employed a time-lag design with a 45-day gap between data collection stages to address CMV. [53]. The paper consisted of three sections: The initial section included questions about the RKH, CPs, and participants’ demographics, covering aspects such as email ID, age, gender, job position, and schooling level. The second section focused on DMQ, while the third addressed SMP. A web link to the questionnaire was shared across 19 pages simultaneously, and administrators requested that it be reshared to motivate members to participate. Data collected via the web link were securely added to a Microsoft Excel sheet, accessible only to the authors. For a sufficient sample size, the study followed the “ten-times approach,” aiming for a minimum of 270 participants (27*10 by [54]). After the initial link sharing, 589 participants provided their feedback. Each respondent’s email ID was connected to an exclusive identification code. Following a 45-day interval, the 2nd section (DMQ) was emailed to the 589 participants, with 401 completing it. The 3rd section (SMP) items were shared with 401 respondents. However, 348 respondents completed the 3^rd^ section of the instrument. Table 1 presents the attrition rates for all three sections of the instruments.

**Table 1:** Data attrition level.

Instrument Sections	Section Shared to	Responses Received	Responses Missed	Attrition Level
Section 1: RKH, CPs, and Data of the Contributors	19 Approved Pages	589	--	--
Section 2: DMQ	589	401	188	31.92%
Section 3: SMPs	401	348	53	13.22%

**Note**: RKH: Rationalized Knowledge Hiding, CPs: Communication Patterns, SMPs: Sensemaking Processes, DMQ: Decision Making Quality.

The attrition rate may be due to participants not regularly logging in to social media platforms or to other assignments. Thus, the early and late responses were compared, and no difference was found [55]. Such measures and outcomes support the reliability and validity of the data.

In addition to testing for CMV/bias, the authors added other tests, namely the Harman single-factor test [56]. When implementing Harman’s single-factor test, all items for the constructs were entered into SPSS, and the resulting options were analyzed. These factors were obtained from an unrotated solution, in which all items of the constructs were used in the principal component factor analysis. Altogether, the total effect of all variables is 37. 110%. The first one explained 13 per cent of the total variance, and it is ideal because the variance should not exceed 50% of the total collective effect [57,58]. Thus, most of the covariances between the exogenous and criterion variables cannot be explained by any of the factors included in the study. Consequently, based on the analyses carried out above, CMV was not discovered in this data set. The demographic profile of the sample group, including the gender, age, qualification, experience, and organizational position of 348 study participants, is provided in S1 Appendix.

### Measurement of variables

*Rationalized knowledge hiding:* RKH is measured with a 04-item instrument developed by [4] at a 5-point Likert scale, where 1 is linked to strongly disagree, and 5 is linked to strongly agree. A sample item is “*Told him/her that my boss would not let anyone share this knowledge”*. Cronbach’s alpha value is 0.827.

*Communication Patterns:* The original scale of CPs, based on 35 items and three sub-scales (Constructive Communication, Mutual Avoidance, and two demand/withdrawal), was developed by [59]. Later, [60] revised the scoring and reliability of the CPs scale. Given the present study’s objective, the authors used items from the Constructive Communication Scale to measure the CPs. [60] provided 09 items, of which 03 were reverse-scored. It asked leaders/managers/supervisors how they and their teams typically deal with workplace issues. Please rate your opinion on a scale of 1 = very unlikely to 5 = very likely. The sample item is: “My team and I try to discuss the problem.” One item was deleted from the final model due to a < 0.5 outer loading value. Cronbach’s alpha value is 0.859.

*Sensemaking Process:* [8] explained the concept of sensemaking in organizations and explained its seven dimensions, namely “Social, Ongoing, Retrospective, Enactment, Cognitive, Identity, and Social Context”. Based on these seven dimensions, a 7-item instrument measures the SMPs. For instance, *“My team members share their interpretations of events”*. However, one item was deleted due to a significantly lower outer loading value. A 5-point Likert scale, where 1 is linked to strongly disagree, and 5 is linked to strongly agree. Cronbach’s alpha value is 0.886.

*Decision-Making Quality:* DMQ was measured with a 7-item instrument developed by [61]. It asked the leaders/managers/supervisors, “How true do you think these statements are true about your decision-making? For instance, ‘searches for three or more choices’. A 5-point Likert scale, where 1 is linked to ‘not very true’ and 5 is linked to ‘very true’. Cronbach’s alpha value is 0.890.

### Statistical approach

There are various statistical models and Software Packages through which one can estimate causal models, and some of the most common are Mplus, AMOS, and SmartPLS. Partial Least Squares Structural Equation Modeling (PLS-SEM) is used to assess this study’s model. PLS-SEM is a globally acknowledged and highly integrated second-generation technique [62]. Through this method, researchers can sum up latent variables measured indirectly by their respective indicators [63]. In model evaluation, co-covariance-based SEM is used to evaluate the developed models. On the other hand, PLS-SEM is used to accept or reject a theory, but may also extend the theory [64]. Thus, the present use of PLS-SEM advances the specification and expansion of theory [62], opined that this method is helpful in exploratory and confirmatory research. PLS-SEM involves a two-step process: the application of the initial measurement model and the evaluation of the structural model. It is used for handling complex models, e.g., mediation and moderation, and is more suitable for small data sets [64]. Smart PLS 4, a veteran software, is operationalized for data analysis.

## Results and analyses

### Model measurements

This study model incorporates four variables and 27 items. Initially, the model’s basic measurement is performed. The model’s reliability is evaluated using Cronbach’s alpha, rho_a, and rho_c. According to [65], Cronbach’s alpha, rho_a, and rho_c values should not less than 0.7. Table 2 confirms that all values exceed 0.7. Convergent validity of the model is also ensured through the average variance extracted (AVE), item reliability, and composite reliability (CR) [62]. According to the experts, the values for AVE, CR, and factor loadings for each variable should be higher than 0.5, 0.7, and 0.7, respectively [62]. Table 2 shows that all AVEs, CRs, and factor loadings meet the required levels. Hence, both reliability and convergent validity have been attained.

**Table 2:** Model measurement.

Variables	Items	Outer Loading	Cronbach’s alpha (α)	CR (rho_a)	CR (rho_c)	AVE
Rationalized Knowledge Hiding (RKH)	RKH-1	0.876	0.827	0.838	0.887	0.666
	RKH-2	0.876				
	RKH-3	0.844				
	RKH-4	0.647				
Communication Patterns (CPs)	CPs-1	0.717	0.859	0.863	0.891	0.507
	CPs-2	0.779				
	CPs-3	0.763				
	CPs-4	0.781				
	CPs-5	0.593				
	CPs-6	0.677				
	CPs-7	0.711				
	CPs-8	0.654				
Sensemaking Processes (SMPs)	SMPs-1	0.859	0.886	0.891	0.898	0.640
	SMPs-2	0.846				
	SMPs-3	0.821				
	SMPs-4	0.798				
	SMPs-5	0.722				
	SMPs-6	0.742				
Decision-Making Quality (DMQ)	DMQ-1	0.838	0.890	0.892	0.891	0.677
	DMQ-2	0.833				
	DMQ-3	0.838				
	DMQ-4	0.859				
	DMQ-5	0.735				
	DMQ-6	0.835				
	DMQ-7	0.815				

Heterotrait–Monotrait (HTMT) ratio analysis and the Fornell–Larcker criterion validate the discriminant validity [65]. As per the Fornell–Larcker technique, the Average Variance Extracted (AVE) square root for each variable should be greater than the correlations with other variables in a similar column [62,66]. Table 3 demonstrates that discriminant validity has been established according to these recommendations, as the top values in each column are consistently higher than those in the other columns. Experts suggest that HTMT ratios less than 0.85 are fine; however, ratios up to 0.90 are acceptable [62]. Table 3, HTMT values are below 0.85 except for one value, 0.890. However, it is also less than 0.90. Hence, it confirms the model’s discriminant validity using both approaches. The inter-construct correlations between some of them are relatively strong, but the discriminant validity is still achieved since the square root of AVE of each construct is higher than the correlations existing between the constructs, as recommended by Hair et al. [67] We also determined HTMT, the desirable criterion, and the maximum value was 0.890, which is less than the 0.90 cutoff [65,67].

**Table 3:** Discriminant validity.

Fornell-Larcker Criterion					Heterotrait-Monotrait Ratio (HTMT)				
	CPs	DMQ	RKH	SMPs		CPs	DMQ	RKH	SMPs
CPs	0.712				CPs				
DMQ	0.707	0.823			DMQ	0.791			
RKH	0.670	0.670	0.816		RKH	0.802	0.767		
SMPs	0.708	0.813	0.650	0.800	SMPs	0.808	0.890	0.753	---

**Note**: RKH: Rationalized Knowledge Hiding, CPs: Communication Patterns, SMPs: Sensemaking Processes, DMQ: Decision Making Quality

**Fig 2:**
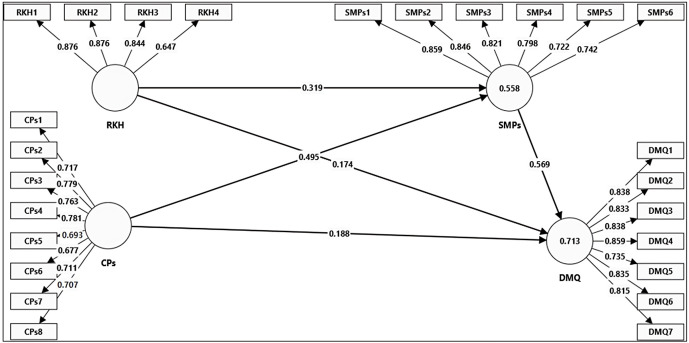
Post-analyses model.

R^2^ is the proportion of the variance in the independent variable explained by the dependent variable [67]. A high R^2^ value greater than 0.5 is a very high correlation between the model variables, mainly when results originate from primary data collection [62]. As illustrated in Fig 2, the R^2^ values for the dependent variable (SMP). In this regard, the reliability of the items is acceptable, with reliability coefficients of self-esteem (α = 0.558) and DMQ (α = 0.713) greater than 0.5 when used as measures to operationalize primary data, indicating a robust model. Q2 is another criterion for confirming the model fit and evaluating whether the conceptual model map matches the actual research map. The Q^2^ values for dependent variables are greater than 0, proving the appropriateness and relevance of the model [62]. Another method for addressing potential collinearity in the data set is the Variance Inflation Factor (VIF). According to specialists’ advice, a VIF below 3 is preferable, but up to 5 is acceptable. All VIF values range from 1.121 to 2.212 and are within the acceptable limit of 3. Therefore, no issue of collinearity is observed for these variables [62]. SRMR value is also under the limit of 0.08 [62].

### Hypotheses confirmation (direct relationship)

SmartPLS 4 (a bootstrapping approach) is used to assess the significance of the hypotheses. Ten thousand samples with replacement are used to operationalize this study’s hypotheses, employing the PLS-SEM model [62]. Table 4 shows that RKH (β = 0.174, p < 0.05) and CPs (β = 0.188, p < 0.05) have significant, positive relationships with DMQ. However, CPs have a stronger association with DMQ than RKH. Thus, H1 and H2 are positively accepted.

**Table 4:** Hypotheses measurement (direct impact).

Structural Paths	Path co-efficient (t-value)	Confidence Interval (95%)	f^2^ Effect Size	(p-value) 0.05	Results
RKH ◊ DMQ	0.174 (3.633)	(0.083-0.272)	0.052	0.000	H1, Accepted
CPs ◊ DMQ	0.188 (4.115)	(0.099-0.277)	0.053	0.000	H2, Accepted
RKH ◊ SMPs	0.319 (5.500)	(0.204-0.433)	0.127	----	Non-Hypothesized
CPs ◊ SMPs	0.495 (9.066)	(0.387-0.602)	0.306	----	Non-Hypothesized
SMPs ◊DMQ	0.569 (11.325)	(0.465-0.661)	0.499	----	Non-Hypothesized

**Note**: RKH: Rationalized Knowledge Hiding, CPs: Communication Patterns, SMPs: Sensemaking Processes, DMQ: Decision Making Quality.

### Hypotheses Confirmation (indirect relationship)

The current study operationalized the “Variance Accounted For” (VAF) method to assess the mediation effects [50]. According to this method, a VAF value higher than 80% indicates complete mediation, a value between 20% and 80% signifies partial mediation, and a value less than 20% denotes no mediation [68]. SMPs are operationalized as a mediator in this study. The SMPs, as mediators, significantly mediate the relationship between RKH, CPs, and DMQ. Table 5: As a mediator, SMPs partially/complementary (VIF = 50.99%) mediate the relationship between RKH and DMQ. Similarly, SMPs also partially mediate (VIF = 60.00%) the relationship between CPs and DMQ. VIF values indicate that SMP is a more effective mediator between CPs and DMQ than RKH and DMQ. Hence, H3 and H4 are accepted. Fig 2 presents the post-analyses of the study.

**Table 5:** Hypotheses measurement (indirect impact).

Indirect Paths	Direct effect, (t-value)	Indirect Effect (t-value)	Total Effect (t-value)	VAF%	Interpretation	Results
RKH **◊**SMPs **◊** DMQ	0.174 (3.633)	0.181 (5.460)	0.355 (6.417)	50.99	Complementary/Partial Mediations	H3, Accepted
CPs **◊**SMPs **◊** DMQ	0.188 (4.115)	0.282 (6.756)	0.470 (9.311)	60.00	Complementary/Partial Mediations	H4, Accepted

**Note**: RKH: Rationalized Knowledge Hiding, CPs: Communication Patterns, SMPs: Sensemaking Processes, DMQ: Decision Making Quality

## Discussion

In this research, the author examines the connection between RKH and CPs, focusing on the mediating effect of SMPs and DMQ. The results from the empirical investigation fully support all four hypotheses in explaining the relationships within IT firms.

Thus, the most interesting finding is the positive link between RKH and DMQ. Knowledge hiding, which is often presented in a rationalized context and commonly viewed as a hostile act, can, under certain circumstances, improve DMQ. This goes against one’s expectations: if managers and supervisors receive information and decide not to share it, then information overload is avoided, and only relevant information is considered. It helps decision-makers focus on relevant and beneficial information, providing accurate, precise insights by avoiding distractions. This aligns with the adage that not all information should be released to the public; sometimes, withholding certain information can help resolve complications.

The particular form of knowledge hiding, namely RKH, can be examined under the umbrella of strategic knowledge management, where knowledge is abundant and, in many cases, overwhelming, becoming a critical factor in discerning relevance. Those managers and supervisors who engage in RKH filter the information that is supposed to reach the decision-makers. This practice not only strengthens the DMQ but also reduces the workload on decision-makers, helping them avoid overloading with irrelevant information. Likewise, the positive correlation between CPs and DMQ confirms the importance of communication in organizations. Since all parties are actively participating, the information flow is straightforward. This helps ensure that all the organization’s stakeholders have a clear understanding of the organization’s environment, objectives, and policies. The CPs facilitate information sharing among managers and supervisors, thereby improving DMQ. Such a conclusion implies that there is a need to continue encouraging communication between the formal organizational decision-makers and the key stakeholders to enhance the overall DMQ, as supported by the literature [27,60,69].

Scholars have identified specific attributes that are evident in effective communication. Firstly, they are characterized by the use of simple language that creates few opportunities for misinterpretations and thus makes several messages understandable. Secondly, they encourage open and transparent communication, which helps prevent the creation of information islands. Thirdly, they are also designed to ensure the timeliness and relevance of information to decision-making agents. Therefore, by cultivating these attributes, people within organizations can establish a communication climate that enhances the quality of the decisions being made [70].

Another contribution of this study is the mediating role of SMPs in the relationships among RKH, CPs, and DMQ. Sensemaking involves taking a continuous feed of information and creating stories that inform decision-making. The results show that SMPs facilitate managers and supervisors in interpreting the information they receive, where it may be selectively ‘muted’ or openly ‘shared’. By doing so, they can interpret the data, discover its meaning, and make more informed and high-quality decisions. This means that sense-making is a continuous process in which organizational members attempt to interpret and communicate information to make sense of existing organizational changes.

The leaders highlight SMPs as critical in making decisions in environments that are shifting and non-linear. SMPs include information sense-making, which encompasses the processing of information; information narrative, which is the ability to build a story from the information; and finally, dynamic adjustment, which is the ability to update the entire process as new information becomes available. In this way, managers and supervisors can gain a deeper understanding of the organizational environment, including its setting, trends, and patterns, and, as a result, make informed decisions. Hence, such types of decisions are compatible with the objectives and goals set for an organization. Therefore, the role of the SMPs, as hypothesized in this study, is to improve the quality of decisions made.

Thus, in general, the present research contributes to understanding the positive associations between RKH, CPs, and DMQ with SMPs as a mediator. Therefore, these findings extend the existing literature by incorporating sensemaking theory [8] with knowledge management and communication theories [27,71,72]. Based on the findings above, the theoretical contributions developed for both the IT industry and academia help intensify and underline the strategic knowledge management role, enhance practical communication practices, and improve sensemaking to enhance DMQ in IT firms. Therefore, by implementing the above practices, organizations can pave the way for effective decision-making and enhance organizational performance. Communication openness reduces the adverse effects of RKH by restoring missing cues and enabling timely feedback. When teams share rationales, check assumptions, and maintain frequent communication, they can detect gaps created by selective withholding and correct interpretations early. This lowers bias and improves the accuracy of collective judgments. Its mitigating effect is most potent when supported by psychological safety, clear roles, and interdependent tasks.

### Theoretical contributions

This research makes a significant contribution to the knowledge of DMQ in organizational contexts by connecting sensemaking theory with the measures of RKH and CPs. Here are the key theoretical contributions. First, the present investigation, by introducing sensemaking theory into the analysis of DMQ, offers a fresh perspective on how RKH and CPs’ communication processes affect decision-making performance. This integration extends the theory into areas beyond the typical KM process domain, providing additional perspectives on the work. Second, this paper enriches insight into RKH by illustrating their moderation on DMQ based on the theory of sensemaking. This contradicts the widely held view that knowledge hiding is inherently harmful, particularly when no justification is provided. Third, the research demonstrates that the flow of CPs directly affects the quality of decisions. This highlights the importance of communication in enhancing the outcomes of organizational choices, while also advancing the theoretical understanding of its role in managerial decision-making. Fourth, by demonstrating that SMPs mediate the relationships among RKH, CPs, and DMQ, this research contributes to understanding the interactions among these factors. It also shows a compelling need to employ sensemaking in converting knowledge and communication into informed decisions. In summary, this research extends the theoretical model of DMQ and reveals that SMPs represent key antecedents of RKH and CPs in the decision-making process.

### Managerial implications

The following are the critical implications of this study for managers in IT firms regarding knowledge management and communication measures for enhancing the quality of decisions: The following implications are particularly noteworthy. *First,* there is an explicit call for managers to recognize that knowledge hiding occurs in organizations and to examine the consequences on DMQ that are fostered through sensemaking processes, as RKH is shown to enhance DMQ. In essence, rather than directly purging knowledge hiding, supervisors should seek ways to order and encourage employees to address the issue appropriately. This entails developing structures that enable employees to justify their decisions regarding the transparent sharing of information, while maintaining the necessary information for high-quality decision-making. Second, CPs are positively associated with the quality of decisions reached, and improvements in communication strength need to be established within the organization. There are aspects that managers need to encourage and facilitate, including, but not limited to, both formal and informal communication, such as group meetings, feedback options, and communication technology. Most managers must understand that by improving CPs, information sharing will be more effective, which in turn improves the DMQ.

Third, since SMPs are the middle link between RKH, CPs, and DMQ, managers should ensure flow development programs that enhance employee sensemaking. This involves acquiring skills to construct meaning from ambiguous information and understand the contexts in which it is presented. Education and development activities that focus on critical assessment and evaluation of information context will help employees appraise and assimilate the information provided, ultimately improving the outcomes of decisions. Fourth, last but not least, managers must find a way to meet the need for effective knowledge sharing while considering all the rationalizations for knowledge hiding. A culture of sharing and justified knowledge withholding can be beneficial for improving DMQ. Implement structured team debriefs, open feedback channels, psychological safety training, documented justification protocols, sense-making workshops, peer reviews, and other relevant practices. In these areas, the manager can ensure that they foster an environment that enhances the generation of high-quality decisions and that communication and sound knowledge management strategies remain central to the undertaking.

### Study limitations and future directions

Like other social studies, this study also has its shortcomings. First, the study utilized cross-sectional questionnaire data collected from managers and supervisors of officially representative IT firms, as posted on their social media pages. This sampling method may introduce selection bias, as patients who complete the form are likely more active on social media than the rest. As a result, the study may not accurately represent other managers and supervisors working in various sectors or those with limited involvement in social media platforms. Therefore, future research should aim to collect data from the population using different sampling techniques to enhance the generalizability of the results. Second, RKH and CPs are considered independent variables influencing DMQ through SMP. It is essential to recognize that these are not the only factors that determine the DMQ. This is usually the case, as other factors, such as organizational culture, leadership styles, or technological tools, can also play significant roles.

Third, this study employed SMP as a mediator but did not thoroughly explore the mechanisms or dimensions of sensemaking. Some considerations, such as individual/collective sensemaking or context-related differences in sensemaking, were not discussed. This study area presents opportunities for future research to explore the detailed processes and the extent of change in sensemaking that affect DMQ. Fourth, the research primarily focuses on IT firms, which comprise the population of firms and may have distinct characteristics that influence the study results. The findings may not be generalized to other industries with other organizational structures. Such a study design may also constrain the generalizability of findings across other sectors and/or organizations. Fifth, longitudinal research designs can be used to examine causality and changes over time. Measuring the variables at different time points would provide even more robust evidence of causality and enable the study of temporal dynamics. Sixth, additional factors that can affect DMQ, such as organizational culture, leadership styles, and other technologies, can be explored further in future research. Seven, future research can focus on understanding the nature of the different forms of sensemaking on the DMQ, such as examining the effects of personal and group sensemaking on decision-making in organizations.

## Supporting information

S1 Appendix Respondent’s demographic information. (DOCX)

S1 File Data file DMQ. (XLSX)
