# Supplementary material for: Sensemaking at work: How rationalized knowledge hiding and communication patterns affect decision quality
Source: PLoS One. 2026 Jul 17;21(7):e0353567. doi: 10.1371/journal.pone.0353567 (PMC13379005; doi:10.1371/journal.pone.0353567)
Supplement: S1 Appendix — (DOCX) [file pone.0353567.s001.docx]

**Appendix S1**

**Respondent’s Demographic Information**

**n=348**

| Dimensions |  | Frequency | Percentage |
| --- | --- | --- | --- |
| Gender | Male | 251 | 72% |
|  | Female | 97 | 28% |
| Age | 25 to 35 | 108 | 31% |
|  | 35 to 45 | 164 | 47% |
|  | 45 and above | 76 | 23% |
| Marital Status | Single | 108 | 31% |
|  | Married | 240 | 69% |
| Qualification | Undergraduate/Diploma in IT | 45 | 13% |
|  | Master | 219 | 63% |
|  | PhD | 84 | 24% |
| Designation | Supervisors | 115 | 33% |
|  | Managers | 150 | 43% |
|  | Senior Leaders /Managers | 83 | 24% |
| Experience | 1 to 5 years | 94 | 27% |
|  | 5 to 10 years | 164 | 47% |
|  | More than 10 years | 90 | 26% |
